# Gut Microbiota Metabolite 3-Indolepropionic Acid Directly Activates Hepatic Stellate Cells by ROS/JNK/p38 Signaling Pathways

**DOI:** 10.3390/biom13101464

**Published:** 2023-09-28

**Authors:** Xiaoyan Yuan, Junting Yang, Yuling Huang, Jia Li, Yuanyuan Li

**Affiliations:** 1 Shanghai Institute of Materia Medica, Chinese Academy of Sciences, Shanghai 201203, China; s20-yuanxiaoyan@simm.ac.cn; 2 Zhongshan Institute for Drug Discovery, Shanghai Institute of Materia Medica, Chinese Academy of Sciences, Zhongshan 528400, China; yjt1481@mail.dlut.edu.cn (J.Y.); huangyuling2605@163.com (Y.H.); 3 University of Chinese Academy of Sciences, Beijing 100049, China; 4 School of Life and Pharmaceutical Sciences, Dalian University of Technology, Dalian 116024, China; 5 School of Pharmaceutical Sciences, Southern Medical University, Guangzhou 510515, China

**Keywords:** gut–liver axis, 3-Indolepropionic acid, HSCs, liver fibrosis

## Abstract

There has been a growing interest in studying the communication of gut microbial metabolites between the gut and the liver as liver fibrosis progresses. Although 3-Indolepropionic acid (IPA) is regarded as a clinically valuable gut metabolite for the treatment of certain chronic diseases, the effects of oral administration of IPA on hepatic fibrosis in different animal models have been conflicting. While some mechanisms have been proposed to explain these contradictory effects, the direct impact of IPA on hepatic fibrosis remains unclear. In this study, we found that IPA could directly activate LX-2 human hepatic stellate cells in vitro. IPA upregulated the expression of fibrogenic marker genes and promoted the features associated with HSCs activation, including proliferation and contractility. IPA also increased reactive oxygen species (ROS) in mitochondria and the expression of inflammation-related genes in LX-2 cells. However, when a ROS-blocking agent was used, these effects were reduced. p38 and JNK, the downstream signaling cascades of ROS, were found to be required for the activation of LX-2 induced by IPA. These findings suggest that IPA can directly activate hepatic stellate cells through ROS-induced JNK and p38 signaling pathways.

## 1. Introduction

Liver fibrosis is a common pathological process that occurs in chronic liver diseases, such as chronic viral infection, alcoholic liver disease (ALD), and non-alcoholic steatohepatitis (NASH) [1,2,3,4]. Late-stage liver fibrosis is irreversible and often leads to cirrhosis or hepatocellular carcinoma (HCC). The activation of hepatic stellate cells (HSCs) is known to be a major factor in the development of liver fibrosis in both experimental models and the human liver [5,6,7,8,9]. HSCs are located in the space between liver sinusoidal endothelial cells and maintain a non-proliferative, quiescent phenotype in a healthy liver [5,6,7,8,9]. However, in the injured liver, they become activated and transform into proliferative myofibroblasts [5,6,7,8,9]. Various factors can directly or indirectly activate HSCs during chronic liver diseases. The mechanism of HSC activation is getting increasingly complex as new pathways and mediators are discovered, including autophagy, endoplasmic reticulum stress, oxidative stress, retinol and cholesterol metabolism, epigenetics, and receptor-mediated signals [7,8,9,10,11,12,13,14,15,16]. Signals from resident hepatic cells and inflammatory cells also play a role in modulating HSC activation [2,3]. Recent evidence suggests that the interaction between the gut and the liver, known as the gut–liver axis, plays a significant role in the development of liver disease [17,18]. Specifically, there is a growing interest in the role of microbial metabolites in the bidirectional communication between the gut and the liver during the progression of liver diseases [19,20,21], which suggests that signals from gut cells can also modulate HSC activation and may be a potential target for preventing and treating the progression of hepatic fibrosis.

**Figure 1:**
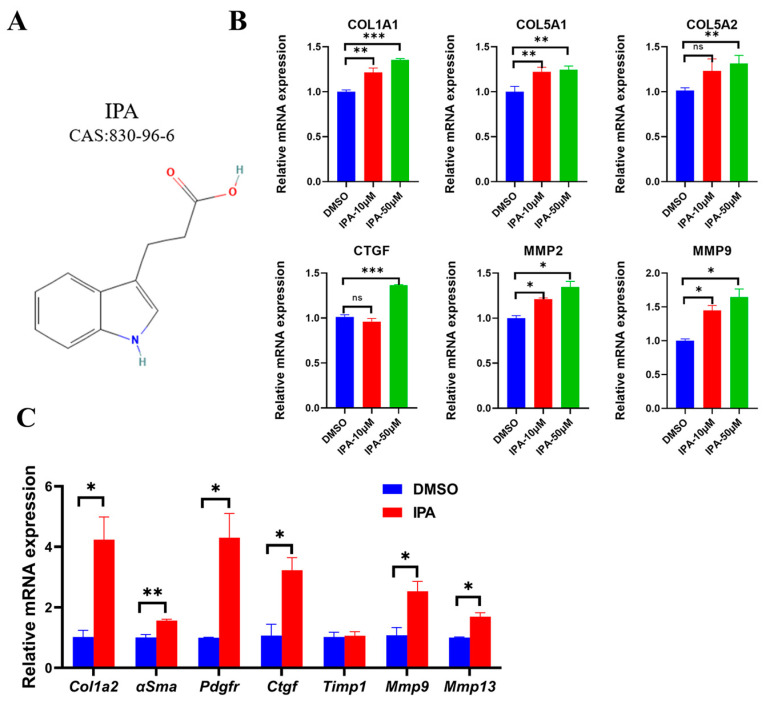
IPA elevated the mRNA expression of fibrogenic genes in HSCs. (**A**) The chemical formula of IPA. (**B**) LX-2 cells were treated with either 10 or 50 µM of IPA for 24 h, the expression of fibrogenic genes (*COL1A1*, *COL5A1*, *COL5A2*, *CTGF*, *MMP2*, *MMP9*) was determined using RT-qPCR. (**C**) Primary mouse hepatic stellate cells were treated with 50 µM of IPA, and the mRNA expression of several fibrogenic genes was determined using RT-qPCR. DMSO represented the control group. Data are shown as mean ± SEM, *: *p* < 0.05, **: *p* < 0.01, ***: *p* < 0.001. Abbreviation: DMSO: Dimethylsulfoxide; IPA: 3-Indolepropionic acid; DAPI: 4,6-diamino-2-phenylindole.

3-Indolepropionic acid (IPA) is a natural compound produced by Clostridium Sporogenes in the gut and metabolized from tryptophan (Figure 1A) [22,23,24]. It is considered to have clinical value in the treatment of chronic kidney disease, Alzheimer’s disease, and cancer [25,26,27,28,29]. However, conflicting effects of the oral administration of IPA on the regulation of hepatic fibrosis have been observed in different animal models of liver injury. In the diet-induced NASH rat model, IPA inhibits the expression of liver fibrogenic and collagen genes, leading to a reduction in hepatic fibrosis [28]. However, in the mouse model of liver damage induced by CCl4, IPA combined with CCl4 worsens liver fibrosis [29]. These conflicting results of IPA on liver fibrosis are influenced by various factors, including diet, genetics, and gut environment, which affect the gut–liver axis [28,29]. Although some mechanisms have been proposed to explain the contradictory effects of oral IPA on liver fibrosis, the direct action of IPA on hepatic fibrosis is still unclear. Understanding this direct action is crucial for the clinical application of IPA in the treatment of liver fibrosis. Therefore, we aim to use the LX-2 human hepatic stellate cell line to evaluate the direct effects of IPA on liver fibrosis and elucidate the potential molecular mechanism.

## 2. Materials and Methods

### 2.1. Cell Culture

The LX-2 human HSC line (Sigma, St.Louis, MO, USA, SCC064) was cultured in Dulbecco’s modified Eagle’s medium-high glucose (DMEM; Gibco, Waltham, MA, USA) supplemented with 10% fetal bovine serum (FBS, Sigma, St.Louis, MO, USA, F8318-500Ml) and 1% penicillin/streptomycin (Gibco, Waltham, MA, USA , 15140-122). The cells were incubated at 37 °C with 5% CO_2_. For the experiment, LX-2 cells were treated with various concentrations (10, 50 µM) of 3-Indolepropionic acid (IPA, Sigma, St.Louis, MO, USA, 220027-1G) for 24 h. Dimethylsulfoxide (DMSO, MP Biomedicals, Irvine, CA, USA, 67-68-5) was used as the control. Additionally, TGFβ1 treatment with a concentration of 5 ng/mL (Peprotech, Rocky Hill, NJ, USA, 100-21) was used as a positive control. In terms of inhibitors, the ROS inhibitor Acetylcysteine (NAC, Selleck, Houston, TX, USA, S1623) was pre-incubated at a concentration of 5 mM for 2 h before adding IPA. The p38 pathway inhibitor SB 202190 (p38i, MCE, Monmouth Junction, NJ, USA, HY-10295) was pre-incubated at a concentration of 20 µM for 2 h before adding IPA. The JNK pathway inhibitor SP600125 (JNKi, MCE, Monmouth Junction, NJ, USA, HY-12041) was pre-incubated at a concentration of 10 µM for 2 h before adding IPA.

### 2.2. MTT Assay

LX-2 cells were placed in 96-well microplates with a density of 8000 cells in 100 µL of culture medium per well and allowed to grow until they became confluent. Then, the LX-2 cells were treated with different concentrations (10, 50 µM) of IPA. After 24 h, the cells were treated with MTT (5 mg/mL, 10 µL per well, Aladdin, T100896-5 g) and incubated at 37 °C for 4 h. The medium in each well was then replaced with 110 µL of DMSO, and the plates were shaken for 10 min. The absorbance was measured at 490 nm. The relative cell viability was expressed as a ratio compared to the cells in the DMSO group.

### 2.3. Gel Contraction Assay

Rat-tail tendon collagen type I (RTTC) was purchased from Corning (Corning, NY, USA, 354,236). Collagen gels were prepared by mixing RTTC, serum-free DMEM, 10 times concentrated PBS, and 1 M NaOH. Then, 300 µL of the mixed collagen gels were added to each well of a 24-well plate and incubated at 37 °C for 30 min to allow them to solidify. After solidification, the gels were gently cut from the 24-well plates using a 10 μL pipette tip. LX-2 cells were detached using 0.25% trypsin, resuspended, and seeded in pretreated 24-well plates. The gels were then treated with IPA for 24 h. Images were captured at 0 h and 24 h. The area of each gel was outlined with a green line. The level of cell contractility was calculated using the formula (Area at 0 h—Area at 24 h) divided by Area at 0 h. The relative cell contractility level was normalized by the DMSO group.

### 2.4. Measurement of Mitochondrial ROS Generation

The production of mitochondrial reactive oxygen species (ROS) was measured using a specific dye called MitoSOX™ Red. The cells were treated with IPA at different concentrations for 24 h and then incubated with a solution containing MitoSOX™ Red for 10 min at 37 °C. After that, the cells were washed with HBSS solution once. Next, the cells were stained with Hoechst 33,342 and washed with PBS. The fluorescent images were immediately observed using a fluorescence microscope. The relative level of mitochondrial ROS was quantified using ImageJ software 1.8.0. nd compared to a control group.

### 2.5. Immunofluorescence (IF)

Immunofluorescence was used to study LX-2 cells. The cells were seeded on chamber slides at a density of 20,000 cells per well in 24-well plates with 1% FBS/DMEM. After 12 h, the cells were treated with IPA (50 µM) and incubated for 24 h. The cells were then fixed with 4% PFA for 10 min at room temperature and permeabilized with 0.1% Triton X-100 in PBS for 10 min. They were then blocked with 3% horse serum for 1 h at room temperature. Primary antibodies (COL1A1, 1:200, Abclonal, Wuhan, China, A1352; Ki67, 1:100, Thermo, Waltham, MA, USA, 11-5698-80) were diluted in 3% horse serum and applied to the chamber slides, which were then incubated overnight at 4 °C. Secondary antibodies were applied for 2 h at room temperature. Coverslips were mounted with Prolong Gold antifade reagent with DAPI (Solarbio, Beijing, China, S2110), and images were captured using a fluorescent microscope. The relative fluorescence level was quantified using ImageJ and normalized to the control group.

### 2.6. Reverse-Transcription-Quantitative PCR (RT-qPCR)

Total RNA was extracted from cells using TRIzol Reagent (Invitrogen, Life Technologies, Tokyo, Japan) following the manufacturer’s instructions. Subsequently, cDNA was synthesized from the total RNA using an RT kit (Promega, Madison, WI, USA). The gene primer sequences were synthesized by Hongxun (Suzhou, China), and qPCR was performed using SYBR Green Supermix (Bio-rad, Hercules, CA, USA). HPRT1 was utilized as the endogenous control gene to account for variations in the total RNA amount in each sample. The expression levels, normalized to HPRT1 levels in each sample, were determined by calculating ΔΔCt. The primer sequences for RT-qPCR can be found in Supplemental Table S1.

### 2.7. Western Blotting

The cells were lysed using NP-40 buffer containing the protease inhibitor PMSF for 30 min on ice. Then, the cells were centrifuged at 12,000 rpm for 15 min at 4 °C. The protein concentration of the supernatant above the sediment was determined using the BCA assay. After all protein samples were mixed with loading buffer and boiled for 10 min, equal amounts of protein (10 μg per well) were subjected to SDS polyacrylamide gel electrophoresis and transferred onto an NC membrane. The membranes were then blocked with 5% non-fat dry skim milk for 1 h at room temperature. After that, the membranes were incubated overnight at 4 °C with the indicated antibodies diluted in TBS-T (Tris-buffered saline containing 0.1% Tween20). The membranes were washed with TBS-T and then incubated for 1 h at room temperature with HRP-conjugated secondary IgGs (CST, Danvers, MA, USA,7074S). The protein bands were visualized using chemiluminescence (ECL blotting reagents, Bio-Rad, Hercules, CA, USA, 1705061). The density of the protein bands was quantified using ImageJ software and normalized to the level of β-tubulin. The following antibodies were used for Western blotting: COL1A1 (1:1000, Abclonal, Wuhan, China, A1352), MMP-2 (1:2000, Proteintech, Wuhan, China, 10373-2-AP), β-tubulin (1:2000, CST, Danvers, MA, USA, 2146S), p38 (1:1000, Abmart, Shanghai, China, T55600), p-p38 (1:1000, Abmart, Shanghai, China, T40076), JNK (1:1000, CST, Danvers, MA, USA, 9252S) and p-JNK (1:1000, CST, Danvers, MA, USA, 9255S).

### 2.8. IPA Treatment of Mouse Primary Hepatic Stellate Cells

Briefly, after in situ perfusion of EDTA, pronase, and collagenase solutions in the anesthetized mouse liver, all cells in the mouse liver were placed in a DNase I, pronase, and collagenase composite solution and subjected to secondary digestion at 125 rpm for 25 min using a constant temperature shaker at 37 °C. Subsequently, density gradient centrifugation was performed using a Nycodenz gradient, and primary mouse hepatic stellate cells were isolated at 4 °C and 1380 g for 17 min. Then, harvest HSCs from the gradient. After the final centrifugation, HSCs were cultured in DMEM supplemented with 10% FBS and 1% penicillin/streptomycin. Three days after the isolation of mouse primary HSCs, cells were treated with IPA (50 µM in DMEM-1% FBS) for 24 h. Subsequently, RT-qPCR was used to detect mRNA expressions of related genes.

### 2.9. Statistical Analysis

The data were analyzed using GraphPad Prism 8.0. Statistical significance was determined using an unpaired Student’s *t*-test. The data are presented as the mean ± standard error of the mean (SEM). A *p*-value less than 0.05 was considered significant. 

## 3. Results

### 3.1. IPA Elevated the mRNA Expression of Fibrogenic Markers of HSCs Activation

We investigated the effect of IPA on the expression of fibrogenic markers in activated HSCs using LX-2 cells. LX-2 cells were treated with IPA at concentrations of 10 μM and 50 μM for 24 h. We then measured the mRNA levels of six fibrogenic genes (*COL1A1*, *COL5A1*, *COL5A2*, *CTGF*, *MMP2*, *MMP9*) in LX-2 cells using RT-qPCR and found the expression of these genes was increased compared to the control group treated with DMSO (Figure 1B), suggesting IPA can directly activate LX-2 cells. Next, we tested whether IPA can also activate primary hepatic stellate cells. Using primary mouse hepatic stellate cells, we found that IPA markedly increased mRNA expression of several hallmark genes of HSC activation, such as *Col1a2*, *αSma*, *Pdgfr*, *Ctgf*, *Mmp9*, and *Mmp13*. These results demonstrate that IPA can activate HSCs from different sources.

### 3.2. IPA Increased the Protein Expression of Hallmark Genes of HSCs

**Figure 2:**
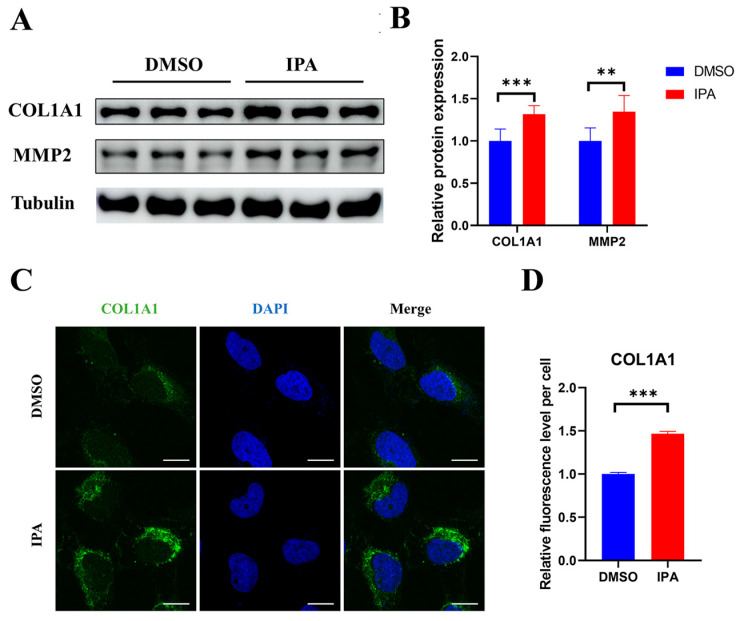
IPA increased the Protein Expression of hallmark genes in LX-2 cells. (**A**) LX-2 cells were treated with 50 µM of IPA for 24 h, the protein expression of COL1A1 and MMP2 was determined using Western blots. Β-tubulin was used as a reference protein (original images can be found in Supplementary Material). (**B**) The quantitative analysis of protein was performed using ImageJ software, and the result was normalized to β-tubulin. (**C**) LX-2 cells were treated with 50 µM of IPA for 24 h, and the expression of COL1A1 protein was determined using immunofluorescence. Green represents COL1A1, blue represents the nuclear. Scale bar, 10 µM. (**D**) The quantitative analysis of fluorescence intensity using ImageJ software and the result was normalized to the control group. Data are shown as mean ± SEM, **: *p* < 0.01, ***: *p* < 0.001. Abbreviation: DAPI: 4,6-diamino-2-phenylindole.

The protein expression levels of COL1A1 and MMP2 were also significantly increased as tested by Western blot (Figure 2A,B). Immunostaining further confirmed the increased expression of the key fibrogenic gene COL1A1 in LX-2 cells after treatment with IPA (Figure 2C,D). Additionally, MTT results showed that IPA at concentrations of 10 and 50 μM did not cause toxicity to LX-2 cells (Supplementary Figure S1). Overall, the increased expression of these fibrogenic genes suggests that IPA directly activates HSCs.

### 3.3. IPA Promoted the Proliferation of HSCs

**Figure 3:**
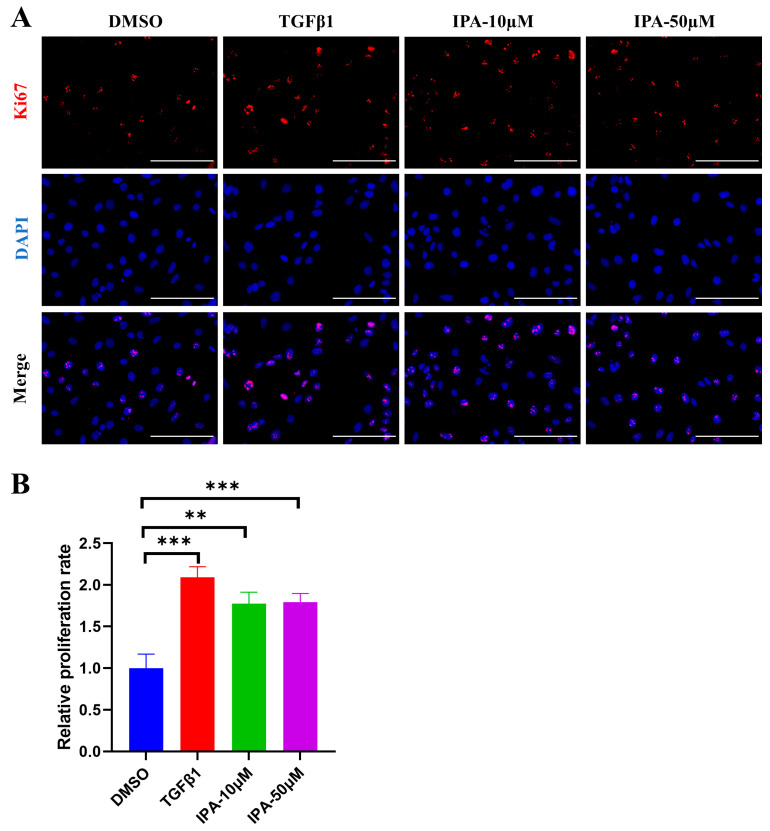
Effects of IPA on the proliferation of LX-2 cells. (**A**) LX-2 cells were incubated with either 10 or 50 µM of IPA for 24 h, and the levels of cell proliferation were determined using the Ki67 staining. TGFβ treatment at 5 ng/mL was used as a positive control. The red represents Ki67 protein, blue represents the nuclear. Scale bar, 100 µM. (**B**) The relative proliferation rate is calculated by dividing Ki67 positive cell numbers by total cell numbers, and the result was normalized to the DMSO-treated group. Data are shown as mean ± SEM, **: *p* < 0.01, ***: *p* < 0.001.

Having seen increased fibrogenic gene expression stimulated by IPA, we further checked whether other features associated with HSC activation were also elevated, such as proliferation. The proliferation of LX-2 was measured using a Ki67 assay. After 24 h of treatment, the percentage of Ki67 positive cells in the IPA-treated LX-2 cells was significantly higher than that of the negative control group treated with DMSO and was comparable to that of the positive control group treated with TGFβ1 (Figure 3A,B). This result demonstrated that IPA could directly promote the proliferation of LX-2, similar to TGFβ1.

### 3.4. IPA Increased Contractility of HSCs

**Figure 4:**
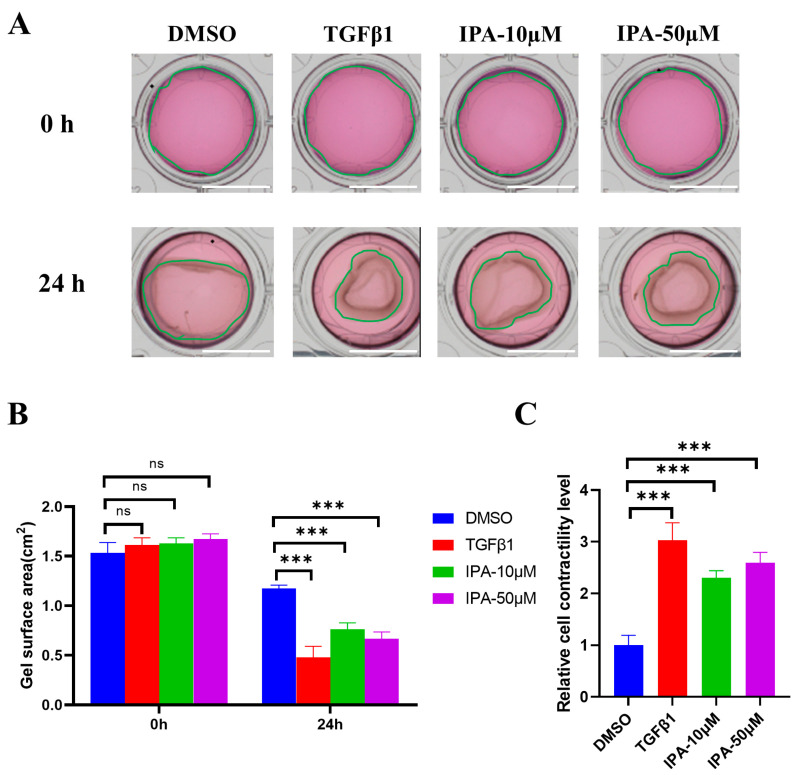
Effects of IPA on the contractility of LX-2 cells. (**A**) LX-2 cells were incubated with either 10 or 50 µM of IPA for 24 h. The levels of cell contractility were determined using gel contraction assay. Treatment with TGFβ1 (5 ng/mL) was used as a positive control. The green line shows the edge of the gel. Scale bar, 1 cm. (**B**) The quantitative analysis of gel surface area at 0 h and 24 h. (**C**)The quantitative analysis of the cell contractility in (**A**). The level of cell contractility was calculated by (Area (0 h)-Area (24 h))/Area (0 h), and the result was normalized to DMSO-treated group. Data are shown as mean ± SEM, ***: *p* < 0.001, ns means no significant differences.

Enhanced contractility is an important feature of activating hepatic stellate cells. Gel contraction assay was used to measure the contractility of LX-2 cells after IPA treatment. As shown in Figure 4A,B, after 24 h of IPA treatment, the gel surface area in the IPA and TGFβ1 treated groups was much smaller than that of the DMSO-treated group, indicating that IPA enhanced the contractility of LX-2 cells similar to the treatment with TGFβ1 (Figure 4C). A similar result was obtained with IPA treatment for 12 h (Supplementary Figure S2). These findings further confirmed that IPA can directly activate HSCs.

### 3.5. IPA Increased Mitochondrial ROS in Activated HSCs

**Figure 5:**
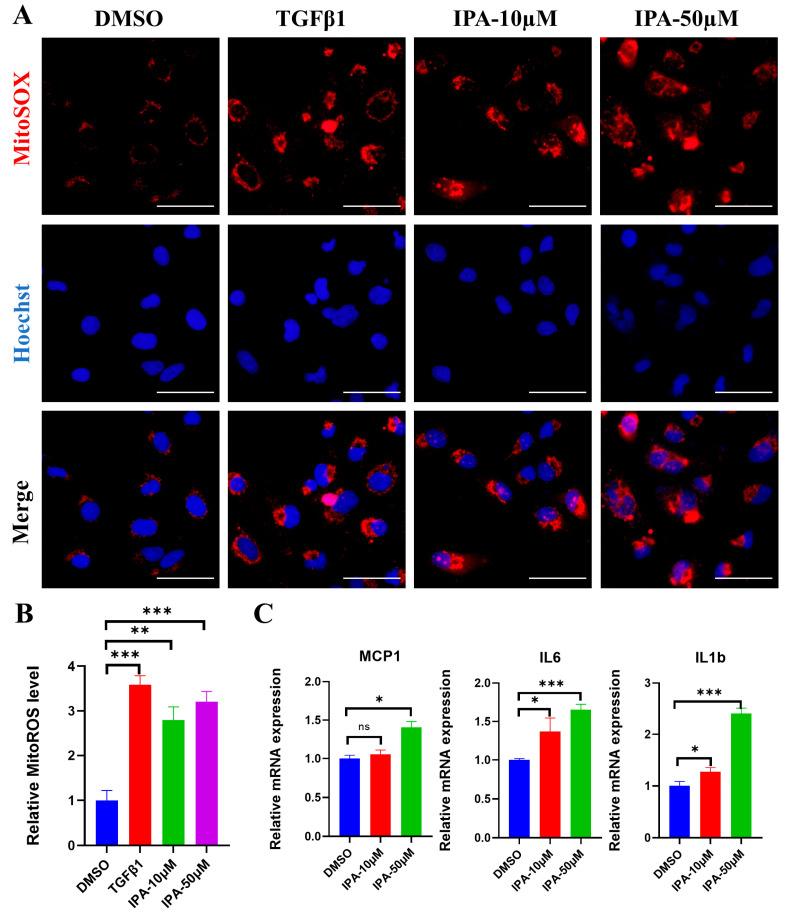
IPA increased mitochondrial ROS in activated LX-2 cells. (**A**) LX-2 cells were incubated with either 10 or 50 µM of IPA for 24 h, mitochondrial ROS levels were determined using MitoSOX. TGFβ1 (5 ng/mL) treated group was used as a positive control. Red represents mitochondrial ROS, and blue represents the cell nuclear. Scale bar, 50 µM. (**B**) The fluorescence intensity of MitoSOX was normalized to the control group. (**C**) The expression of inflammation genes (*MCP1*, *IL6*, *IL1b*) associated with ROS were determined using RT-qPCR. Data are shown as mean ± SEM, *: *p* < 0.05, **: *p* < 0.01, ***: *p* < 0.001, ns means no significant differences.

The generation of reactive oxygen species (ROS) has been found to be closely linked to the activation of hepatic stellate cells (HSCs) [8]. Previous studies have reported that IPA has different effects on oxidative stress depending on the conditions [23,26]. In order to investigate the direct effect of IPA on oxidative stress in HSCs, the levels of mitochondrial ROS in LX-2 cells were measured after treatment with IPA at concentrations of 10 and 50 μM using MitoSOX red (Figure 5A). The results showed that IPA increased the production of mitochondrial ROS by almost three folds (Figure 5B). The expression of inflammatory genes associated with oxidative stress in LX-2 cells was also determined using RT-PCR after treatment with IPA. The mRNA levels of *MCP-1*, *IL-6*, and *IL1b* were increased dose-dependently when treated with IPA (Figure 5C).

### 3.6. ROS Scavenger Blocked the Activation of HSCs Induced by IPA

**Figure 6:**
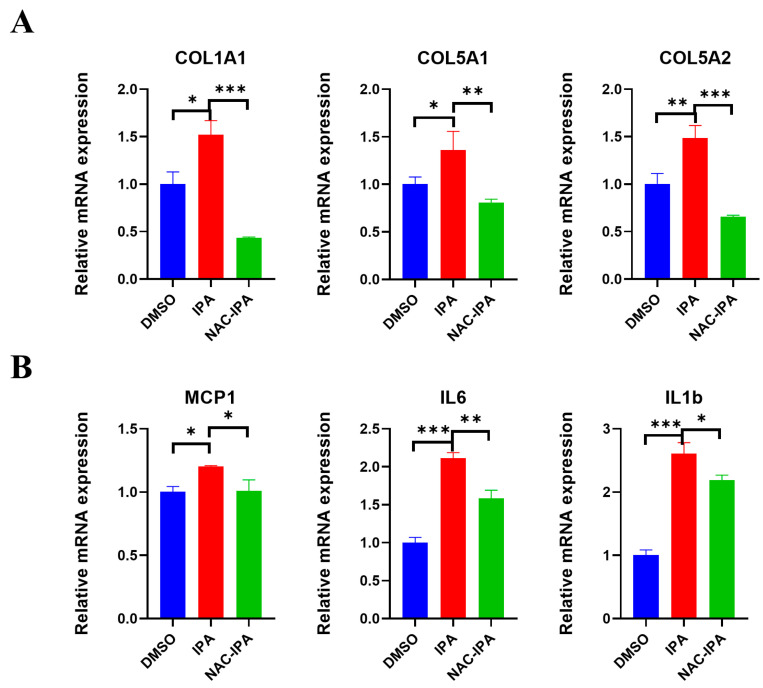
Blocking ROS pathway attenuated IPA-induced activation of LX-2 cells. LX-2 cells were pre-incubated with NAC(Acetylcysteine) for 2 h, and then treated with 50 μM IPA for 24 h. (**A**) the mRNA levels of the fibrogenic genes (*COL1A1*, *COL5A1*, *COL5A2*) and (**B**) the inflammatory genes (*MCP1*, *IL6*, *IL1b*) associated with ROS were measured using RT-qPCR. Data are shown as mean ± SEM, *: *p* < 0.05, **: *p* < 0.01, ***: *p* < 0.001.

Next, we used NAC, a ROS-blocking agent, to determine the impact of IPA on HSC activation. LX-2 cells were incubated with NAC for 2 h before being treated with IPA for 24 h. The expression of five fibrogenic genes that were previously upregulated was significantly inhibited (Figure 6A, Supplementary Figure S3), and the expression of the oxidative stress-related genes triggered by IPA (Figure 5C) was also downregulated (Figure 6B). These results indicate that IPA directly activates HSCs via the ROS pathway.

### 3.7. IPA Treatment Activates p38 and JNK Signaling Pathways in HSCs

**Figure 7:**
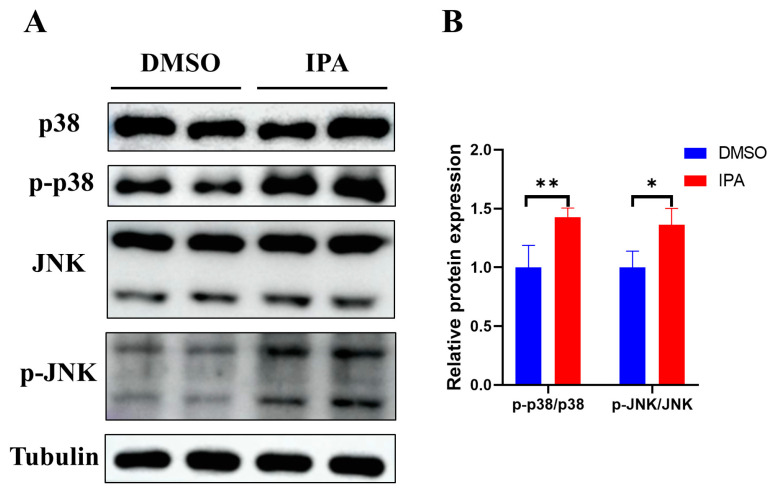
IPA activated p38 and JNK pathways in LX-2 cells. (**A**) LX-2 cells were treated with 50 µM IPA for 24 h, the phosphorylation status and the expression levels of p38 and JNK were determined using Western blots. Tubulin was used as a reference protein. (**B**) The quantitative analysis of protein was performed using ImageJ software, and the result was normalized to Tubulin. *n* = 4. Data are shown as mean ± SEM, *: *p* < 0.05, **: *p* < 0.01.

Next, we investigated the impact of p38 and JNK activation, two classic signaling cascades downstream of ROS, on the activation of LX-2 cells induced by IPA. As shown in Figure 7, treatment with 50 μM of IPA for 24 h markedly increased the phosphorylation levels of both p38 and JNK in LX-2 cells. These results indicate that IPA activates p38 and JNK signaling pathways in HSCs.

### 3.8. Both p38 and JNK Are Required for IPA-Induced ROS Production in Activated HSCs

**Figure 8:**
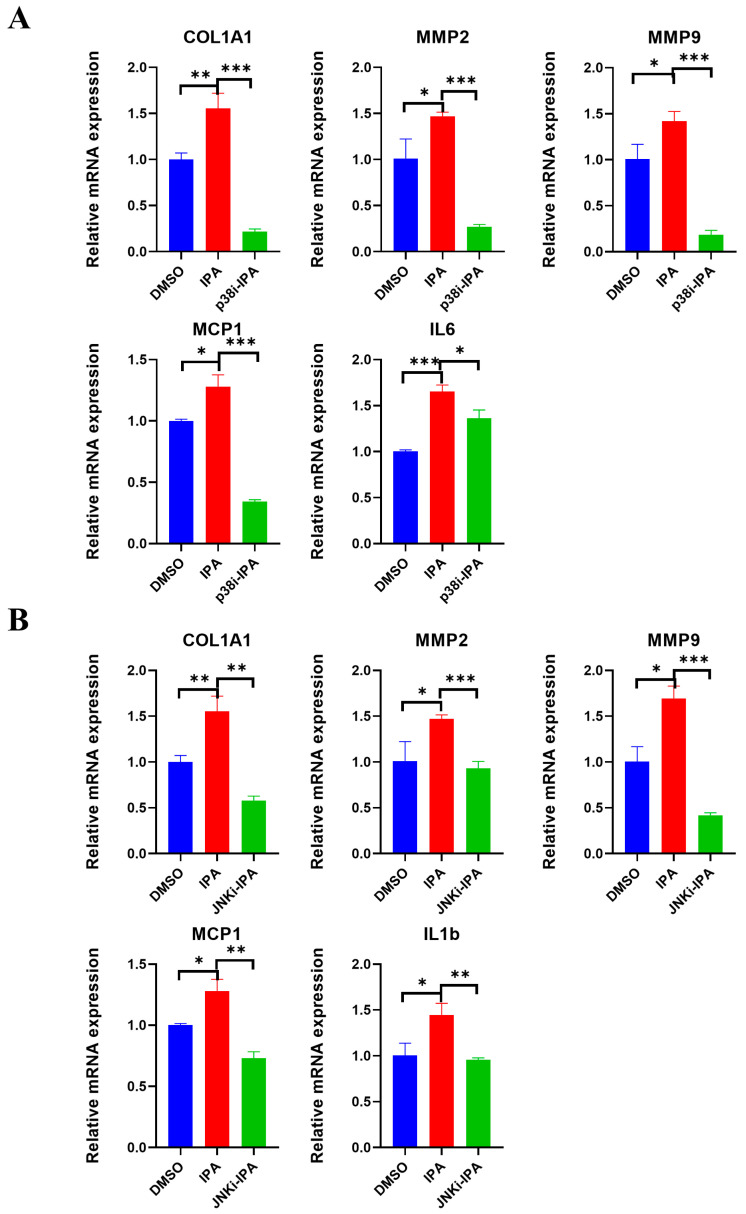
Both p38 and JNK are required for IPA-induced ROS production in LX-2 cells. LX-2 cells were pre-incubated with either (**A**) p38 inhibitor SB 202190 (20 µM) or (**B**) JNK inhibitor SP600125 (10 µM) for 2 h, and then treated with IPA (50 µM) for 24 h, the RNA levels of the fibrogenic genes (*COL1A1*, *MMP2*, *MMP9*) and inflammatory genes (*MCP1*, *IL6*, *IL1b*) were determined using RT-qPCR. Data are shown as mean ± SEM, *: *p* < 0.05, **: *p* < 0.01, ***: *p* < 0.001.

Next, we investigated the impact of p38 and JNK activation, two classic signaling cascades downstream of ROS, on the activation of LX-2 cells induced by IPA. To investigate whether the activation of p38 and JNK are crucial to the HSC activation stimulated by IPA, inhibitors of these two pathways were added together with IPA. The p38 inhibitor prevented the increase in the expression of inflammatory genes, IL-6 and MCP1 (Figure 8A). Similarly, the JNK inhibitor prevented the increase in the expression of inflammatory genes, MCP1 and IL-1b (Figure 8B). Additionally, both inhibitors were able to downregulate the expression of fibrogenic genes, *COL1A1* and *MMP2*, and *MMP9* in LX-2 cells treated with IPA. These results suggest that both p38 and JNK are required to activate HSCs induced by IPA.

## 4. Discussion

The gut–liver axis has been found to play a significant role in liver fibrosis modulation. The metabolites produced by the gut microbiota act as signaling molecules between the gut and liver, exerting bidirectional effects on the gut–liver axis [17,18]. Understanding how these metabolites affect the gut and liver is crucial for the treatment of liver fibrosis by targeting the gut–liver axis [17,18,19,20,21]. IPA, a metabolite of tryptophan by intestinal microbiota, was reported to have various effects in different chronic diseases [22,23,24,25,26,27,28,29]. However, its effects on liver illnesses, such as liver fibrosis, have only lately been reported [28,29]. While IPA attenuates NASH-induced fibrosis [28], it exacerbates CCl4-induced fibrosis [29]. Both studies were conducted on animals given IPA orally without examining the direct effect of IPA on hepatic stellate cells, the master cells that drive liver fibrosis development. It is critical to explore the direct effects of IPA on hepatic stellate cells and elucidate the underlying mechanisms in order to guide future proper use of IPA in treating liver fibrosis. A previous study reported that IPA combined with TGF-β group exhibited a reduction in the expression of fibrosis-related genes compared to the TGF-β group, but it is important to note that this is not a direct effect of IPA on HSC activation [30]. In our study, using LX-2, a commonly used human hepatic stellate cell lines [31], we find that IPA directly activates LX-2 through the ROS/JNK/p38 signaling pathway. We observed that IPA, even at a relatively low concentration (10 µM), was able to activate LX-2 cells, as shown by increased fibrogenic gene expression, gel contraction capacity, and proliferation rate. Our findings support the previous study that IPA oral administration can deteriorate CCl4-induced fibrosis [29] and provide another conceivable explanation for how IPA might directly stimulate hepatic stellate cells and cause liver fibrosis in vivo. Regarding the opposite effects observed in NASH animals, as reported previously [21], there are several possible explanations. Firstly, IPA can directly act on hepatocytes and attenuate lipid accumulation [32], thus mitigating lipotoxicity-triggered liver inflammation and fibrosis, as observed in IPA NASH study. Secondly, IPA was found to augment GLP-1 secretion from intestinal L cells [33]. GLP-1 can attenuate liver steatosis, inflammation, and fibrosis through multiple mechanisms, such as reducing appetite and body weight [34], as it is observed in the NASH study. Therefore, our findings are important, which agree with CCl4 induced-fibrosis study, providing guidance for the further clinical application of IPA for the treatment of liver fibrosis. Since IPA may only improve NASH-induced liver fibrosis and may exacerbate liver fibrosis from other etiologies, such as HBV or HCV, it is important to take these etiologies into consideration while using IPA.

According to our findings, IPA increases hepatic stellate cell activation via the ROS-induced MAPK pathway. ROS is an essential signaling transmitter in hepatic stellate cells, promoting activation, migration, and proliferation [8,35,36]. IPA has been shown to have varying effects on oxidative stress depending on the circumstance [22,23,24]. In this study, we found that a low concentration of 10 μM of IPA increased mitochondrial ROS in LX-2 cells. A similar result was observed in 4T1 breast cancer cells, where IPA enhanced the production of mitochondrial reactive species [26]. However, when IPA was used at a high concentration of more than 1 mM, it showed a protective effect against lipid oxidative stress in hepatic microsomes, preventing iron-induced oxidative damage to cell membranes [37]. These results suggest that the various effects of IPA on oxidative stress may be dependent on the dosage as well as its location inside cells. In particular, IPA protects against lipid oxidative stress in microsomes at high concentrations while largely increasing ROS in mitochondria at low doses. In this study, we selected two concentrations of IPA, namely 10 and 50 μM, strategically chosen to align with the range of IPA blood concentrations observed in clinical trials (NCT01898884). The concentration of 50 μM closely approximates the average serum concentration of IPA, while the 10 μM concentration surpasses the fundamental physiological serum level of approximately 1 µM [38]. By including data from these two concentrations, our study aims to provide valuable insights and constructive information relevant to the clinical application of IPA.

The MAP kinases p38 and JNK are known to be the key signaling pathways activated by oxidative stress [39]. ROS activates p38 and JNK in different ways in different cells [40,41,42,43]. Our study showed that blocking p38 and JNK individually could prevent IPA from activating LX-2 cells. This finding implies that p38 and JNK are both required for IPA-induced stress. Inhibiting p38 and JNK also reduced inflammation caused by ROS, as shown by reduced mRNA expression of inflammatory genes, such as *IL1b*, *IL6*, *MCP-1*, etc. This prevents further activation of hepatic stellate cells by these inflammatory cytokines. Targeting both p38 and JNK could thereby counteract IPA-induced hepatic stellate cell activation.

## 5. Conclusions

This study demonstrates that IPA can directly activate fibrogenic processes in LX-2 human hepatic stellate cells at a relatively low concentration. The ROS-induced p38/JNK signaling pathway is responsible for this activation. The in vitro findings can contribute to our understanding of how oral IPA can modulate liver fibrosis through the gut–liver axis and provide insights into potential applications for IPA in preclinical and clinical settings in the future. It should also be concluded that results demonstrate a potential profibrogenic effect of IPA at supra-physiological concentrations after exogenous administration but not at physiological concentrations.

## Data Availability

The data used to support the findings of this study are available from the corresponding author upon reasonable request.

## Supplementary Materials

The following supporting information can be downloaded at: https://www.mdpi.com/article/10.3390/biom13101464/s1, Figure S1: Effects of IPA on the cell viability of LX-2. LX-2 cells were treated with either 10 or 50 µM IPA for 24 h, the cell viability was determined using MTT assay. DMSO as the control group. *n* = 4. Data are shown as mean ± SEM, ns means no significant differences.; Figure S2: Effects of IPA on the contractility of LX-2 cells. (A) LX-2 cells were incubated with either 10 µM or 50 µM of IPA for 12 h. The levels of cell contractility were determined using gel contraction assay. Treatment with TGFβ1 (5 ng/ml) was used as a positive control. The green line shows the edge of the gel. Scale bar, 1cm. (B) The quantitative analysis of gel surface area at 0 h and 24 h. (C) The quantitative analysis of the cell contractility in (A). The level of cell contractility was calculated by (Area(0 h)-Area(12 h))/Area(0 h) and the result was normalized to DMSO treated group. Data are shown as mean ± SEM, **: *p* < 0.01, ***: *p* < 0.001. ns means no significant differences. Figure S3: Inhibition of ROS pathway downregulated fibrogenic genes *CTGF* and *MMP9*. LX-2 cells were pre-incubated with NAC(Acetylcysteine) for 2 h, and then treated with IPA for 24 h, the RNA levels of *CTGF* and *MMP9* were detected using RT-qPCR. *n* = 3. Data are shown as mean ± SEM, *: *p* < 0.05, **: *p* < 0.01. Table S1. Primers for RT-qPCR.
