# Supplementary material for: Gut Microbiota Metabolite 3-Indolepropionic Acid Directly Activates Hepatic Stellate Cells by ROS/JNK/p38 Signaling Pathways
Source: Biomolecules. 2023 Sep 28;13(10):1464. doi: 10.3390/biom13101464 (PMC10604901; doi:10.3390/biom13101464)
Supplement: Supplementary file 1 [file biomolecules-13-01464-s001.zip › biomolecules-2580950-supplementary.pdf]

Supplementary Information for

Gut Microbiota Metabolite 3-Indolepropionic Acid Directly Activates Hepatic Stellate Cells by ROS/JNK/p38 Signaling Pathways

Xiaoyan Yuan[1,2,3†], Junting Yang[2,4†], Yuling Huang[2,5], Jia Li[1,2,3,5*], Yuanyuan Li[1,2,3,5*]

[1] Shanghai Institute of Materia Medica, Chinese Academy of Sciences, Shanghai 201203, China; s20-yuanxiaoyan@simm.ac.cn (X.Y.)

[2] Zhongshan Institute for Drug Discovery, Shanghai Institute of Materia Medica, Chinese Academy of Sciences, Zhongshan 528400, China; yjt1481@mail.dlut.edu.cn; huangyuling2605@163.com (Y.H.)

[3] University of Chinese Academy of Sciences, Beijing 100049, China

[4] School of Life and Pharmaceutical Sciences, Dalian University of Technology, Dalian 116024, China

[5] School of Pharmaceutical Sciences, Southern Medical University, Guangzhou 510515, China

* Correspondence: Jia Li, jli@simm.ac.cn; Yuanyuan Li, liyuanyuan@simm.ac.cn

† These authors contributed equally to this work

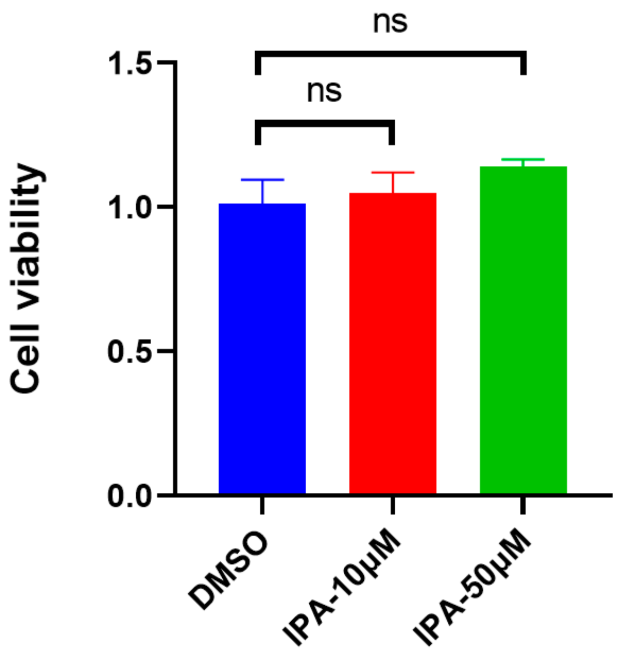

Supplemental Figure S1. Effects of IPA on the cell viability of LX-2. LX-2 cells were treated with either 10μM or 50μM IPA for 24h, the cell viability was determined using MTT assay. DMSO as the control group. n=4. Data are shown as mean ± SEM, ns means no significant differences.

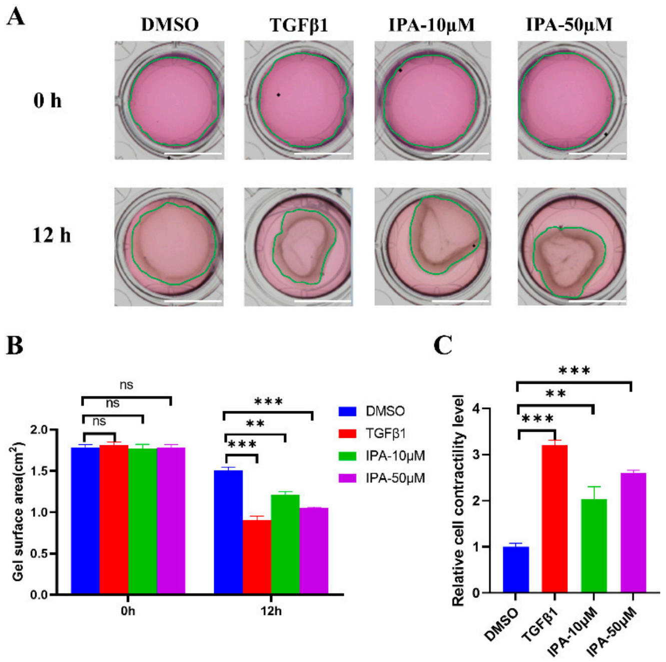

Supplemental Figure S2. Effects of IPA on the contractility of LX-2 cells. (A) LX-2 cells were incubated with either 10 µM or 50 µM of IPA for 12 h. The levels of cell contractility were determined using gel contraction assay. Treatment with TGFβ1 (5 ng/ml) was used as a positive control. The green line shows the edge of the gel. Scale bar, 1cm. (B) The quantitative analysis of gel surface area at 0 h and 24 h. (C) The quantitative analysis of the cell contractility in (A). The level of cell contractility was calculated by (Area(0 h)-Area(12 h))/Area(0 h) and the result was normalized to DMSO treated group. Data are shown as mean ± SEM, **: $p < 0.01$, ***: $p < 0.001$. ns means no significant differences.

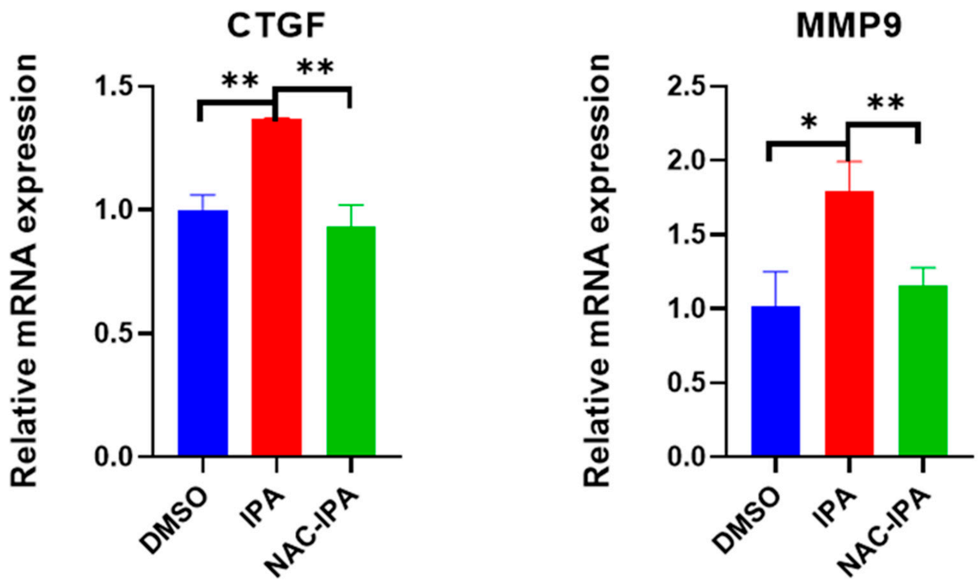

Supplemental Figure S3. Inhibition of ROS pathway downregulated fibrogenic genes *CTGF* and *MMP9*. LX-2 cells were pre-incubated with NAC(Acetylcysteine) for 2h, and then treated with IPA for 24h, the RNA levels of *CTGF* and *MMP9* were detected using RT-qPCR. n=3. Data are shown as mean± SEM, *: $p < 0.05$, **: $p < 0.01$.

Gene	Forward sequence	Reverse sequence
COL1A1	ATCCCACCAATCACCTGCGT	TCATCGCACAACACCTTGCC
COL5A1	GGAGATGATGGTCCCAAAGGCA	CCATCATCTCCTTTGTCACCAGG
COL5A2	CAGGCTCCATAGGAATCAGAGG	CCAGCATTTCCTGCTTCTCCAG
CTGF	ATGGTGCTCCCTGCATCTTC	GTTTGGTCCTTGGGCTCGTC
MMP2	TCCCATTTTGATGACGATGA	CCGTACTTGCCATCCTTCTC
MMP9	CGGCCACTACTGTGCCTTT	GCGATGGCGTCGAAGATG
MCP1	GACCCCAAGCAGAAGTGGGT	GTGTCTGGGGAAAGCTAGGGG

IL6	TTCCAAAGATGTAGCCGCCC	GATGCCGTCGAGGATGTACC
IL1b	TCCTTTCAGGGCCAATCCCC	GGGAGCGAATGACAGAGGGT
HPRT1	TGCTGAGGATTTGGAAAGGGTGTTT	GCACACAGAGGGCTACAATGTGATG

Supplemental Table S1. Primers for RT-qPCR.
